# The immunogenic cross-talk in pediatric MASLD: does subclinical immune dysfunction accelerate early hepatic destruction?

**DOI:** 10.3389/fimmu.2026.1930909

**Published:** 2026-09-17

**Authors:** Harald Mangge

**Affiliations:** Clinical Institute of Medical and Chemical Laboratory Diagnostics, Medical University of Graz, Graz, Austria

**Keywords:** common variable immunodeficiency, gut-liver axis, intestinal barrier, metabolic endotoxemia, pediatric MASLD

## Abstract

The pathogenesis of pediatric Metabolic Dysfunction-Associated Steatotic Liver Disease (MASLD) is increasingly recognized as an immunometabolic process driven by chronic low-grade inflammation (metainflammation). While nutritional surplus and visceral adiposity are established primary drivers, the contribution of underlying subclinical host immune variations remains largely unexplored. This mini-review investigates the intersection between childhood obesity and subclinical humoral immune dysfunctions, specifically variants of Common Variable Immunodeficiency (CVID). We propose that subtle defects in adaptive immunity—such as partial secretory IgA or IgG subclass deficiencies—compromise mucosal barrier integrity, triggering a breakdown of the gut-liver axis. This barrier failure allows an influx of pathogen-associated molecular patterns (PAMPs) into the portal circulation, overwhelming hepatic immune tolerance. Within the liver, this persistent microbial exposure primes Toll-like receptor pathways and activates the NLRP3 inflammasome within Kupffer cells, accelerating hepatocyte pyroptosis and tissue damage. Concurrently, the T-cell dysregulation characteristic of CVID variants interacts with hepatic lipotoxicity, changing the disease phenotype from simple steatosis to aggressive tissue remodeling. Identifying these subclinical immunodeficiencies in progressive pediatric MASLD is essential for developing personalized, targeted immunometabolic therapies for at-risk youth.

## 1 Introduction

The rising prevalence of pediatric Metabolic Dysfunction-Associated Steatotic Liver Disease (MASLD) reflects the global childhood obesity epidemic (1). Once viewed as a chronic metabolic complication of adulthood, progressive hepatic inflammation and tissue injury are now frequently diagnosed in children and adolescents. The recent nomenclature change from non-alcoholic fatty liver disease (NAFLD) to MASLD highlights that hepatic fat accumulation is fundamentally linked to systemic metabolic and endocrine dysregulation. However, metabolic overnutrition alone does not fully explain why some children develop aggressive hepatocyte destruction and rapid fibrosis while others maintain stable, simple steatosis (1).

Emerging research indicates that the rate of disease progression is determined by the immune system’s response to metabolic stress (2). Pediatric MASLD is now understood as an immunometabolic continuum, where continuous lipid overload triggers an innate immune response within the liver. This metainflammation turns resident macrophages (Kupffer cells) and endothelial cells into active drivers of tissue damage (2). While initial research has focused on nutrient-derived danger signals, the role of pre-existing, subclinical variations in host immunity remains an important area of study, which operates alongside established risk factors like high-fructose diets and genetic susceptibility (e.g., *PNPLA3* rs738409 variants) (17).

A key focus of this mini-review is the potential impact of subclinical Common Variable Immunodeficiency (CVID) variants (3). CVID is characterized by impaired B-cell differentiation and hypogammaglobulinemia, but subclinical variants often present with mild immunoglobulin deficits or selective antibody deficiencies that can escape early clinical diagnosis (3). Traditionally, CVID-related liver complications, such as nodular regenerative hyperplasia or granulomatous infiltrates, were studied independently of metabolic factors (4). This mini-review explores how childhood obesity can act as a metabolic trigger that compounds subclinical immune dysfunction, accelerating early liver damage through increased mucosal permeability, hyper-reactive innate immune pathways, and altered T-cell regulation (4, 5).

## 2 The broken frontier: gut-liver axis breakdown in subclinical immunodeficiency

The gut-liver axis serves as the primary barrier regulating systemic exposure to intestinal antigens (5, 6). In the context of combined childhood obesity and subclinical CVID variants, this barrier system experiences dual metabolic and immunological stress, accelerating early hepatic injury.

### 2.1 The mucosal shield deficit: impaired secretory IgA and dysbiosis

Secretory IgA (sIgA) is the primary immunoglobulin protecting mucosal surfaces, responsible for binding and neutralizing intestinal pathogens and preventing their adherence to the epithelial wall (7). Subclinical CVID variants often involve low sIgA levels. This impairment disrupts mucosal immune exclusion, allowing opportunistic bacteria to colonize the mucus layer (7, 8).

In childhood obesity, diets high in saturated fats and refined sugars alter the gut microbiota, reducing beneficial short-chain fatty acid (SCFA)-producing species and increasing Gram-negative proteobacteria (9). While these microbial shifts are well-documented in pediatric observational cohorts, mechanistic validation predominantly stems from murine models, demonstrating that when combined with an sIgA deficiency, this dysbiosis worsens, leading to bacterial overgrowth and increased production of immunogenic bacterial components within the intestinal lumen (8, 9).

### 2.2 Epithelial barrier failure and uncontrolled translocation

Under physiological conditions, enterocyte integrity is maintained by tight junction protein complexes, including zonula occludens-1 (ZO-1), occludin, and claudins (10, 11). Experimental data from rodent models indicates that the systemic inflammation associated with obesity triggers mucosal TNF-α production, which activates myosin light chain kinase (MLCK) and is proposed to accelerate the internalization and degradation of these tight junctions (11). Lacking the protective effects of sIgA and structural tight junctions, the intestinal epithelium becomes abnormally permeable (10, 14). This structural failure permits the unregulated paracellular translocation of viable bacteria, bacterial fragments, and pathogen-associated molecular patterns (PAMPs) directly into the lamina propria (14).

### 2.3 Portal endotoxemia and overwhelmed hepatic tolerance

Translocated antigens bypass local mesenteric lymph nodes and travel directly through the portal vein to the liver (12). This exposure is proposed to accelerate chronic portal endotoxemia, characterized by elevated levels of circulating lipopolysaccharides (LPS), peptidoglycans, and unmethylated Cytosine–phosphate–Guanine (CpG) bacterial DNA entering the hepatic sinusoids (12).

The healthy pediatric liver maintains immune tolerance to low levels of portal antigens through anti-inflammatory signaling from sinusoidal endothelial cells and resting Kupffer cells (19). However, the high concentration of PAMPs resulting from a combined metabolic and immunodeficient state overwhelms these homeostatic mechanisms (5). This persistent exposure shifts the hepatic microenvironment toward a pro-inflammatory state, priming innate immune receptors and initiating early-stage tissue injury (5, 6).

## 3 Hyper-inflammatory innate cascades: TLR4 priming, NLRP3 hyper-activation, and clinical laboratory staging

The persistent influx of intestinal PAMPs through the compromised portal vein triggers a destructive innate immune cascade inside the liver (12). In children where obesity is combined with a subclinical CVID variant, this pathway is hypothesized to become hyper-reactive, drawing from molecular mechanisms established in experimental models of endotoxemia.

### 3.1 Innate hyper-reactivity: TLR4 priming and the MyD88 cascade

*In vitro* and *in vivo* functional models demonstrate that when lipopolysaccharides (LPS) and related bacterial endotoxins reach the hepatic sinusoids, they bind to Toll-like Receptor 4 (TLR4) complexes on resident Kupffer cells and sinusoidal endothelial cells (5). This interaction requires the co-receptors myeloid differentiation factor 2 (MD-2) and CD14. Binding triggers the canonical MyD88-dependent signaling pathway, recruiting interleukin-1 receptor-associated kinases (IRAK1/4) and tumor necrosis factor receptor-associated factor 6 (TRAF6).

This cascade is proposed to accelerate the phosphorylation and degradation of IκB, allowing the transcription factor nuclear factor kappa B (NF-κB) to translocate into the cell nucleus. NF-κB activation induces the transcription of pro-inflammatory cytokines, including Tumor Necrosis Factor-alpha (TNF-α) and Interleukin-6 (IL-6). Crucially, it provides the essential “Signal 1” for inflammasome activation by upregulating the intracellular synthesis of inactive pro-caspase-1, pro-interleukin-1 beta (pro-IL-1β), and pro-interleukin-18 (pro-IL-18).

### 3.2 The inflammasome trigger: NLRP3 hyper-activation and tissue loss

In this primed state, the liver is highly sensitive to metabolic stressors, such as altered, hydrophobic bile acids (e.g., deoxycholic acid [DCA]) and saturated free fatty acids (SFAs) (13, 14). As shown in mechanistic cellular assays, these lipotoxic molecules provide Signal 2, which drives the physical assembly of the inflammasome (14). These metabolic stressors cause lysosomal destabilization, reactive oxygen species (ROS) production, and potassium (K^+^) efflux from the cytoplasm. These cellular changes trigger the oligomerization of NLRP3 (NLR family pyrin domain containing 3) with the adaptor protein ASC (apoptosis-associated speck-like protein containing a Caspase Recruitment Domain [CARD]) and pro-caspase-1.

In subclinical CVID variants, the immune system often lacks adequate regulatory control loops, such as IL-10 signaling or functioning T-regulatory cell feedback. As a result, the assembly of the NLRP3 complex is uninhibited. Active caspase-1 cleaves pro-IL-1β and pro-IL-18 into their active, inflammatory forms. Simultaneously, it cleaves gasdermin D (GSDMD). Characterized extensively in preclinical models of steatohepatitis (16), the N-terminal fragments of gasdermin D translocate to the hepatocyte plasma membrane, oligomerizing to form large pores. This is proposed to accelerate pyroptosis—an inflammatory cell death where the cell swells and bursts, releasing active cytokines and toxic lipids into the parenchyma, which accelerates tissue loss (16).

### 3.3 Clinical laboratory screening: identifying subclinical CVID in pediatric MASLD

Because subclinical CVID variants do not present with classic hypogammaglobulinemia (such as a total collapse of IgG), specialized diagnostic criteria are required to identify these at-risk pediatric patients (3, 4).

When a child with obesity exhibits progressive liver enzyme elevation that does not respond to standard metabolic lifestyle modifications, the following diagnostic screening panel, derived from international clinical immunology (ESID) criteria for atypical humoral defects (3), should be evaluated (1, 17):

#### 3.3.1 Total and subclass quantitative immunoglobulins:

- Serum IgG: Often sits at the lower limit of normal (LLN) for age (e.g., 5.0 - 6.5 g/L in older children), rather than showing an overt deficiency (3).
- IgG Subclass Titers: Marked deficiencies in IgG2 or IgG4 subclasses provide early evidence of B-cell maturation deficits (3).
- Serum IgA: Value < 0.7 g/L (or complete selective IgA deficiency < 0.07 g/L). This is a critical indicator of compromised mucosal immunity and a weakened gut-liver barrier (7).

#### 3.3.2 Functional antibody competence (vaccine response)

- Evaluation Method: Measuring specific IgG antibody titers against protein antigens (Tetanus/Diphtheria) and polysaccharide antigens (*Streptococcus pneumoniae*).
- Pathological Cut-off: An inability to mount or maintain a protective antibody response following standard immunization protocols (defined as a < 4-fold rise in post-booster titers). This functional deficit confirms a subclinical humoral immune defect, even when total IgG levels appear normal (3).

#### 3.3.3 Advanced flow cytometry (peripheral B-cell phenotyping)

- Memory B-Cell Fractions: A significant reduction in switched memory B-cells (CD19^+^CD27^+^IgD^−^IgM^−^) to < 2% of total circulating B-lymphocytes.
- Significance: This reduction indicates a defect in the germinal center reaction, a key feature of CVID variants (3, 4). It identifies patients prone to systematic immune dysregulation and hyper-inflammatory macrophage activation. **Table 1** summarizes the pathophysiologic cascade of combined metabolic and immunologic gut-liver axis stress.

**Table 1:** Pathophysiological cascade of combined metabolic and immunological gut-liver axis stress.

Stage/process	Pathophysiological mechanism	Clinical explanation & impact	Evidence source & model type	Key references
1. Dual Stress Trigger	Childhood Obesity + Subclinical CVID	The baseline condition where metabolic stress from obesity coexists with an underlying primary immunodeficiency.	Pediatric Cohorts (Obesity) & Adult/Pediatric Data (CVID)	(2, 3)
2a. Metabolic Pathway	High-fat/sugar intake	High caloric intake alters intestinal tight junctions, is hypothesized to amplify microbial dysbiosis, and compromises gut wall integrity.	Preclinical Models (Diet-induced) & Human Observational	(9, 11)
2b. Immunological Pathway	Deficient secretory IgA (sIgA)	The immune deficit leads to a loss of mucosal immune exclusion and bacterial overgrowth, removing the gut’s natural protective shield.	Human Immunology Data (CVID-derived)	(7, 8)
3. Barrier Breakdown	Severe epithelial barrier breakdown	The convergence of metabolic damage and immune failure is hypothesized to amplify a complete collapse of the intestinal lining.	Preclinical Models & *In Vitro* Barrier Assays	(10, 11)
4. Translocation	Portal endotoxemia (Influx of PAMPs)	Uncontrolled translocation allows Pathogen-Associated Molecular Patterns (like LPS and bacterial DNA) to leak into the portal vein.	Preclinical *In Vivo* Models (Metabolic Endotoxemia)	(12)
5. Hepatic Impact	Overwhelmed hepatic tolerance	PAMPs are transported directly to the liver, oversaturating its detoxification capacity and triggering early tissue injury.	Preclinical Models (Mechanistic) & Adult Cohorts (NASH)	(5, 6)

### 3.4 Current state of direct pediatric evidence and translational gaps

While the entire longitudinal cascade connecting subclinical humoral defects directly to accelerated hepatic pyroptosis has not yet been tracked within a single pediatric cohort, distinct steps are clinically validated in youth. Pediatric primary immunodeficiency studies confirm that low sIgA and IgG subclass deficits drive early mucosal barrier failure and gut dysbiosis. Separately, serum assays in children with progressive MASLD consistently demonstrate elevated lipopolysaccharide (LPS) and soluble CD14 levels, confirming active portal endotoxemia. Finally, immunohistochemical analyses of pediatric liver biopsy cores have confirmed intrahepatic TLR4 upregulation, active NLRP3 inflammasome assembly, and gasdermin D-mediated hepatocyte pyroptosis in aggressive pediatric MASH phenotypes. Gathering these individual clinical pieces underpins the validity of the proposed immunometabolic sequence in at-risk youth.

## 4 T-cell dysregulation and mixed phenotypes: the emergence of granulomatous MASLD

The transition from silent steatosis to progressive hepatic destruction in the context of subclinical CVID is not solely mediated by innate cascades. It relies also on an altered adaptive immune compartment (4, 15). The hallmark of CVID variants includes systematic T-cell dysregulation, characterized by a contraction of regulatory T-cells (Treg) and an uninhibited polarization toward pro-inflammatory T-helper 1 (Th1) and Th17 phenotypes (15). When this altered lymphocyte pool encounters the lipotoxic environment of childhood obesity, a distinct, aggressive liver phenotype emerges (4, 5).

Under physiological conditions, hepatic CD4^+^CD25^+^FoxP3^+^ Treg cells maintain tissue homeostasis by secreting Interleukin-10 (IL-10) and Transforming Growth Factor-beta (TGF-β), suppressing aberrant auto-reactive or hyper-inflammatory lymphocytic infiltration. In subclinical CVID variants, this protective Treg pool is reduced (15).

Concurrently, resident hepatic dendritic cells, continuously stimulated by translocated portal PAMPs, secrete high levels of IL-12 and IL-23, forcing the differentiation of naive T-cells into Th1 and Th17 effectors. These hyper-reactive T-cells infiltrate the hepatic parenchyma, clustering around the steatotic portal tracks.

The resulting chronic secretion of Interferon-gamma (IFN-γ) by Th1 cells activates Kupffer cells into a highly destructive state, while Th17-derived IL-17 activates hepatic stellate cells directly, accelerating collagen deposition.

This distinct immunological environment alters the presentation of metabolic fatty liver disease. Instead of standard lobular steatohepatitis, these pediatric patients may develop a proposed conceptual phenotype: Granulomatous MASLD (4). The combination of lipid accumulation within hepatocytes and uninhibited macrophage activation leads to the formation of non-caseating epithelioid granulomas scattered throughout the hepatic lobules (4, 15). While clinicians must rule out classic etiologies of pediatric hepatic granulomas—such as sarcoidosis, chronic granulomatous disease, or drug-induced injury—this metabolic-immunological crossover isolates fat droplets and apoptotic debris, accelerating structural destruction of the surrounding parenchyma and triggering early, atypical portal fibrosis in young patients.

## 5 Clinical implications and therapeutic frontiers: dual-targeted immunometabolic regimens

The discovery of a combined subclinical CVID and MASLD phenotype in pediatric obesity changes the approach to clinical staging and therapeutic intervention (4). Relying solely on conventional metabolic therapies will fail to halt disease progression if the underlying adaptive immune defect and gut-barrier failure are left unaddressed (5).

### 5.1 T proposed future strategy: targeted subcutaneous immunoglobulin optimization

For pediatric patients confirmed to have selective antibody failures or low-normal IgG levels coupled with memory B-cell contraction, standard lifestyle modifications should be paired with targeted, personalized immunoglobulin replacement therapy (SCIG or IVIG) (3). While typically reserved for severe clinical infections, low-dose SCIG therapy can help restore circulating IgG pools and support mucosal surfaces. This intervention boosts immune exclusion within the lamina propria, reduces intestinal dysbiosis, and helps halt the translocation of PAMPs into the portal vein, effectively turning off “Signal 1” of the hepatic innate immune cascade (7, 12).

### 5.2 Established clinical practice: metabolic and incretin axis modulation

To address the metabolic drivers of this disease, next-generation incretin-based therapies provide significant benefit:

- GLP-1/GIP Receptor Agonists (e.g., Tirzepatide): These dual-targeting agents address the metabolic drivers by reducing visceral adiposity and decreasing the flow of free fatty acids to the liver (1).
- Reversing Hepatic Insulin Resistance: By improving insulin sensitivity within the liver, these agents restore the liver–alpha-cell feedback loop, reducing the hyperglucagonemia that drives *de novo* lipogenesis and intracellular bile acid retention (18).

### 5.3 Proposed future strategy: intestinal epithelial fortification via biotics

Targeted mucosal therapies are essential for repairing the damaged gut barrier (14). High-dose, multi-strain synbiotic regimens containing *Lactobacillus rhamnosus* GG and specific prebiotics stimulate enterocyte tight junction expression (ZO-1, occludin) (10). This fortification repairs the paracellular pathway, preventing the systemic endotoxemia that drives hepatic metainflammation (12).

## 6 Conclusion and future outlook

Pediatric MASLD is a complex, multi-system disorder where metabolic overload interacts with host immunity (1, 2). This mini-review highlights a critical clinical scenario: the intersection of childhood obesity with subclinical humoral immune dysfunctions, such as CVID variants (4, 5).

When a child experiences both metabolic overnutrition and a subclinical adaptive immune defect, the gut-liver axis breaks down (5). The resulting loss of secretory IgA and tight junction integrity allows an influx of portal endotoxins into the liver, triggering TLR4 priming and NLRP3 inflammasome hyper-activation within Kupffer cells (12). This process drives inflammatory hepatocyte cell death via pyroptosis (16).

Simultaneously, the T-cell dysregulation characteristic of CVID variants drives the formation of an aggressive, mixed granulomatous MASLD phenotype, causing rapid liver injury at a young age (4, 15).

### 6.1 Limitations, clinical risks, and future trial imperatives

The primary limitation of the current literature is the absence of unified, longitudinal pediatric cohorts tracking subclinical humoral immune dysfunction and MASLD simultaneously. Consequently, the proposed sequential cascade—from mucosal sIgA deficiency to hepatic pyroptosis—remains a mechanistic model inferred from separate metabolic and immunological datasets. Furthermore, therapeutic translation carries distinct clinical risks. While GLP-1/GIP agonists are established in pediatric obesity, utilizing SCIG/IVIG to target portal endotoxemia remains an off-label approach for subclinical CVID variants, posing risks of volume overload, localized reactions, or inappropriate resource allocation. Therefore, validating this immunometabolic framework requires prospective, multi-center pediatric trials. These studies must prioritize strict stratification based on vaccine responses and memory B-cell fractions, utilizing non-invasive biomarkers of gut-barrier integrity and hepatic damage before interventional protocols can be widely considered (see **Table 2**).

**Table 2:** Categorization and levels of evidence for the proposed pathophysiological mechanisms linking humoral immune dysfunction and pediatric MASLD.

Pathophysiological process/concept	Level of evidence	Primary source type	Key references
Obesity-induced dysbiosis and tight junction disruption	Established	Pediatric cohort observations & Murine models	(6, 9, 11)
Innate TLR4 priming and NLRP3 activation by PAMPs/lipotoxicity	Established	*In vitro* assays & Pediatric biopsy IHC	(5, 12, 16)
CVID-associated T-cell dysregulation (Th1/Th17 shift, Treg contraction)	Established	Human immunodeficiency clinical data	(15)
Full, sequential causal chain from sIgA deficiency to hepatic pyroptosis	Hypothesis	Proposed model based on separate cohorts	(1, 4)
The ‘Granulomatous MASLD’ Phenotype	Hypothesis	Extrapolated from adult CVID phenotypes	(4, 15)
Efficacy of low-dose SCIG/IVIG therapy to reduce portal endotoxemia	Hypothesis	Proposed future clinical trial framework	(3, 4)

### 6.2 Future outlook

To improve outcomes for this patient population, future research and clinical workflows should focus on two main areas:

#### 6.2.1 Comprehensive immunological screening

Children presenting with progressive MASLD who do not respond to standard metabolic interventions should undergo detailed immunological screening (1, 17). This assessment should include quantitative IgG/IgA subclass measurements, vaccine antibody responses, and memory B-cell flow cytometry to identify underlying immune variants (3, 4).

#### 6.2.2 Stratified clinical trials

Future clinical trials should evaluate combined therapeutic strategies. Investigating protocols that pair mucosal barrier protection (such as targeted immunoglobulins or synbiotics) with metabolic therapies (GLP-1 receptor agonists) will be essential for developing effective, personalized treatments that address both the metabolic and immunological drivers of liver injury in youth.

## References

[1] Furthner D Weghuber D Dalus C Lukas A Stundner-Ladenhauf HN Mangge H . Nonalcoholic fatty liver disease in children with obesity: Narrative review and research gaps. Horm Res Paediatr. (2022) 95:167–76. doi: 10.1159/000525543 34351306

[2] Hotamisligil GS . Inflammation and metabolic disorders. Nature. (2006) 444:860–7. doi: 10.1038/nature05485 17167474

[3] Ballow M . Primary immunodeficiency disorders: Antibody deficiencies. J Allergy Clin Immunol. (2002) 109:581–91. doi: 10.1067/mai.2002.122466 11941303

[4] Nedelea I Nicoara-Farcau O Procopet B Stefanescu H Radu C Balan R . Liver disease in common variable immunodeficiency: Current evidence and knowledge gaps. Int J Mol Sci. (2026) 27:1518. doi: 10.3390/ijms27031518 41683939 PMC12898428

[5] Marra F Svegliati-Baroni G . Lipotoxicity and the gut-liver axis in NASH pathogenesis. J Hepatol. (2018) 68:280–95. doi: 10.1016/j.jhep.2017.11.014 29154964

[6] Tilg H Adolph TE Trauner M . Gut-liver axis: Pathophysiological concepts and clinical implications. Cell Metab. (2022) 34:1700–18. doi: 10.1016/j.cmet.2022.09.017 36208625

[7] Mantis NJ Rol N Corthésy B . Secretory IgA's complex roles in immunity and mucosal homeostasis in the gut. Mucosal Immunol. (2011) 4:603–11. doi: 10.1038/mi.2011.41 21975936 PMC3774538

[8] Macpherson AJ Mccoy KD Johansen FE Brandtzaeg P . The immune geography of IgA in the intestinal lumen. Mucosal Immunol. (2008) 1:11–22. doi: 10.1038/mi.2007.6 19079156

[9] Cani PD Bibiloni R Knauf C Waget A Neyrinck AM Delzenne NM . Changes in gut microbiota control metabolic endotoxemia-induced inflammation in high-fat diet-induced obesity and diabetes in mice. Diabetes. (2008) 57:1470–81. doi: 10.2337/db07-1403 18305141

[10] Chelakkot C Ghim J Ryu SH . Mechanisms regulating intestinal barrier integrity and its pathologic implications. Exp Mol Med. (2018) 50:1–9. doi: 10.1038/s12276-018-0126-x 30115904 PMC6095905

[11] Turner JR . Intestinal mucosal barrier function in health and disease. Nat Rev Immunol. (2009) 9:799–809. doi: 10.1038/nri2653 19855405

[12] Cani PD Amar J Iglesias MA Poggi M Knauf C Bastelica D . Metabolic endotoxemia initiates obesity and insulin resistance. Diabetes. (2007) 56:1761–72. doi: 10.2337/db06-1491 17456850

[13] Fitzinger J Rodriguez-Blanco G Herrmann M Borenich A Stauber R Aigner E . Gender-specific bile acid profiles in non-alcoholic fatty liver disease. Nutrients. (2024) 16:250. doi: 10.3390/nu16020250 38257143 PMC10821077

[14] Chavez-Talavera O Tailleux A Lefebvre P Staels B . Bile acid control of metabolism and inflammation. Gastroenterology. (2017) 152:1679–94. doi: 10.1053/j.gastro.2017.02.048 28214524

[15] Azizi G Hafezi N Mohammadi H Yazdani R Alinia T Tavakol M . Abnormality of regulatory T cells in common variable immunodeficiency. Cell Immunol. (2017) 315:11–7. doi: 10.1016/j.cellimm.2016.12.007 28284485

[16] Wree A McGeough MD Peña CA Inzaugarat ME Canbay A Hoffman HM . NLRP3 inflammasome activation is required for fibrosis development in NAFLD. J Mol Med (Berl). (2014) 92:1069–82. doi: 10.1007/s00109-014-1170-1 24861026 PMC4349416

[17] Mangge H Baumgartner B Zelzer S . Patatin-like phospholipase 3 (rs738409) gene polymorphism is associated with increased liver enzymes in obese adolescents and metabolic syndrome in all ages. Aliment Pharmacol Ther. (2015) 42:99–105. doi: 10.1111/apt.13232 25939720

[18] Maruszczak K Mangge H Weghuber D . Determinants of hyperglucagonemia in pediatric non-alcoholic fatty liver disease. Front Endocrinol. (2022) 13:1004128. doi: 10.3389/fendo.2022.1004128 36133310 PMC9483010

[19] König J Wells J Cani PD García-Ródenas CL MacDonald T Mercenier A . Human intestinal barrier function in health and disease. Clin Transl Gastroenterol. (2016) 7:e196. doi: 10.1038/ctg.2016.54 27763627 PMC5288588

